# Ocular posterior pole pathological modifications related to complicated pregnancy. A review

**DOI:** 10.22336/rjo.2017.16

**Published:** 2017

**Authors:** Vanessa Andrada Păun, Zamfir-Radu Ionescu, Liliana Voinea, Monica Cîrstoiu, Alexandru Baroș, Ștefan Pricopie, Radu Ciuluvică

**Affiliations:** * Ophthalmology Clinic, University Emergency Hospital, Bucharest, Romania; ** Department of Pathology, Pediatric Hospital, Pitești, Romania; Genetics Department, Faculty of Sciences, State University Pitești, Romania; *** Ophthalmology Department, “Carol Davila” University of Medicine and Pharmacy; University Emergency Hospital, Bucharest, Romania; **** Clinic of Obstetrics and Gynecology, University Emergency Hospital, Bucharest; “Carol Davila” University of Medicine and Pharmacy, Bucharest, Romania; ***** Ist Clinic of General Surgery, University Emergency Hospital, Bucharest; “Carol Davila” University of Medicine and Pharmacy, Bucharest, Romania; ****** Ophthalmology Clinic, University Emergency Hospital, Bucharest, Romania; ******* “Carol Davila” Dental Medicine University, Bucharest, Romania

**Keywords:** retina, pregnancy, preeclampsia, eclampsia, hypertension, anti-phospholipid syndrome, central serous chorioretinopathy, Sheehan’s syndrome

## Abstract

Ocular posterior pole modification are a pathological manifestation in complicated pregnancies, especially when pregnancy induced hypertension is present (PIH), as well as in preeclampsia (PE) or eclampsia. Nonetheless, as the pregnancy evolves, the possibility for an aggravated evolution with HELLP syndrome, disseminated intravascular coagulation, and idiopathic thrombocytopenic purpura may have an ocular manifestation that, mainly, implies a loss of visual field or acuity, that, left unattended, may constitute a permanent impairment. Pregestational conditions like pituitary adenoma or genetic pedigree for complement factor H gene (1q31.1) single nucleotide mutations could lead to central serous chorioretinopathy or retinal detachment with severe, ischemic, central cilioretinal artery or vein occlusion and optic nerve atrophy. Furthermore, although subtle in many cases, any new visual symptoms during pregnancy should constitute an alarming factor for obstetrical reevaluation and ophthalmological approach in order to preserve the mother’s quality of life.

## Introduction

Pregnancy is known to cause physiological changes in the parturient. These physiological changes may affect different organs and systems, including the eyes and visual perception. As it is known in modern literature, these changes include physiologic and pathologic modifications, the latter being already significantly influenced by any preexisting condition that may remain undiagnosed prior to the ophthalmological consultation. Thus, regarding the above affirmations, we may subdivide the pathological changes into three categories, related to the time of onset: first time ocular pathology during pregnancy, modification of preexisting known or diagnosed ocular pathology and ocular complications of systemic diseases [**1**]. Certain diseases like Sheenan syndrome, eclampsia and preeclampsia are specific to pregnancy while other diseases like Graves disease, disseminated intravascular coagulation and intracranial hypertension are more likely to have an increased incidence or risk for complications during pregnancy. Furthermore, these diseases require pregestational silent morphological anomalies that undergo significant changes during pregnancy or labor, and may have a significant clinical presentation [**2**,**3**].

**Preeclampsia**

As it is already accepted, preeclampsia (PE) is defined as the presence of a systolic blood pressure, greater than 140 mmHg (SBP ≥ 140 mmHg) or a diastolic blood pressure greater than or equal to 90 mmHg (DBP ≥ 90 mmHg), values measured, in a previous normotensive female patient, on two different consultations that have to be at least 4 hours apart one from the other. In the case of an emergency diagnosis requirement, the SBP must be higher than 160 mmHg (SBP ≥ 160 mmHg) and a diastolic pressure as high as 110 mmHg (DBP ≥ 110 mmHg), occasion that allows immediate antihypertensive therapy [**4**]. Furthermore, impaired hepatic function, noticeable due to elevated liver enzymes concentrations and severe, pharmacologically nonresponsive, epigastric or upper abdominal pain, may become an immediate diagnostic clue. Progressive renal insufficiency, with initial proteinuria greater than 0.3 grams in a 24-hours duration urine specimen, new onset of any neurological disturbances, pulmonary oedema, and thrombocytopenia may help distinguish a general, systemic, multiple organic impairment [**5**]. 

Retinal dysfunction in preeclampsia female patients seems to be the most serious ocular complication, thus, implying fundoscopic examination, because retinal vascular changes are the immediate result of hypertension status, being, also, a mirror for any vascular changes. Therefore, the ophthalmoscopic examination for ocular fundus may become an important screening consultation for different placental anomalies. The most common abnormality identified in the retina of PE patients is represented by arterioles narrowing, that may have visual symptoms when the spasm involved become focal or generalized, accompanied by exudates, hemorrhages, peripapillary or focal retinal edema, serous retinal detachment or, even acute ischemic optic neuropathy with transient visual blindness. Sometimes, complications may evaluate even further to retinal pigment epithelial lesions, severe macula edema with retinal detachment – this step being an emergency as it emerges to permanent blindness, central to retinal artery occlusion and rapid optic atrophy [**6**]. These pathological eventualities may express in 25% of the PE patients and 50% of the eclampsia patients with blurred vision, photopsia, scotomas, diplopia, amaurosis, and dyschromatopsia [**7**]. Cortical blindness associated with PE and pregnancy induced hypertension (PIH) is a documented entity, frequently encountered in a severely decreased visual acuity patient with normal pupillary light response, thus, consisting a differential diagnosis against cortical blindness or a functional symptom. In such situations, brain MRI examination with occipital lobe attenuation becomes confirmatory for the diagnosis. The required treatment implies fast blood pressure control, being the most successful pharmacological factor, as heparin and corticosteroids seem not to be significantly favorable [**8**]. The exudative retinal detachments are rarely seen in PIH or PE patients. The retinal pigmentary epithelium lesions, or Elscching spots, may sometimes be seen in a PE patient with prior choroidal infarcts and visual impairing. Macular edema or papilledema with retinal detachment requires pregnancy termination in order to save the mother’s vision: in this medical issue, ophthalmological surgery being futile [**9**]. Serous retinal detachments are encountered in severe cases of PE, being unconditionally related to a higher degree of choroidal perfusion impairment resulting in a subretinal leakage, sometimes, constituting in serous exudative retinal detachments visible as bullous, bilateral in the mandatory context of a PIH [**6**,**10**]. Although there are no explicit guidelines for the surgical procedures, fundus grade II and III changes (**Table 1**), i.e. hemorrhages and exudates, should be carefully assessed in order to terminate pregnancy trough cesarean section, with a maximum delay of 3 to 4 weeks in favor of fetus maturity. Furthermore, retinal hemorrhage, transudate, papilledema in the context of PIH, retinal detachment should be a sufficient indication for the immediate gestation termination regardless of any delay possibility or fetal age [**10**]. Severe retinopathy changes are correlated with a compromised maternal-fetal circulation. It seems that the progression of retinal vascular changes becomes a sign of increasing severity of PIH, thus, implying a higher mortality rate. Some studies suggested that PIH and fundus changes, in particular, are associated with low birth weight (p < 0.05), a lower 1 min Apgar score (less than 5 points) and in extreme situations, with stillbirth [**11**]. 

**Table 1:** Keith-Wanger-Barker (1939) Classification of hypertensive retinopathy [**27**].

Grade	Classification;
Grade I	Generalized retinal arteriolar narrowing or sclerosis;
Grade II	Focal arterial narrowing; arteriovenous crossings; Moderate – Severe arterial sclerosis; Arterial light reflex – exaggerated;
Grade III	Abnormalities seen in grades I and II; Retinal hemorrhages; Representative exudation; Cotton-wool spots.
Grade IV	Abnormalities seen in grades I-II-III; Optic nerve and macular star swelling

**Central serous chorioretinopathy**

**Fig. 1:**
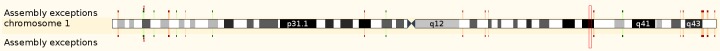
Gene locus of complement H factor 1 on autosomal 1st chromosome (modified chart, from EMBL-EBI e! Chromosome)

**Fig. 2:**
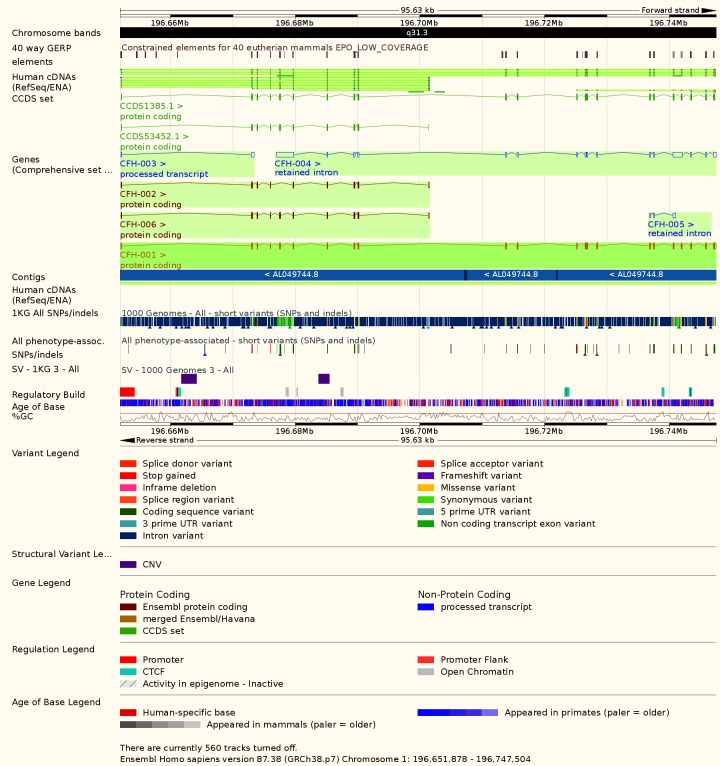
Genomic details of complement H factor gene (1q31.1) associated with familial autosomal dominant and recessive CSR (modified, from EMBL-EBI e! Chromosome).

Central serous chorioretinopathy (CSR) can occur during the first and third trimester. The symptomatology consists in metamorphopsia, visual loss, scotomas, and light sensitivity. The CSR disease is represented by the serous detachment of the neurosensory retina, due to the choriocapillaris leakage at the retinal pigment epithelium occasioned by choroidal neovascularization. Although many mechanisms have been proposed for the CSR etiology, it seems that inflammation is not a key factor, since glucocorticoids rather aggravate than improve CSR. Strong evidence for a genetic contribution to the CSR pathogenesis has been documented in families of CSR patients, although no specific genotype or hereditary pattern has ever been associated with CSR. Genetic studies on single nucleotide mutations have been performed on different cohorts suffering from CSR. It seems that the complement factor H gene, located on the long arm of the first autosomal chromosome (1q31.3 – OMIM *134370, **Fig. 1**,**2**), involved in the macular age related degeneration (OMIM #610698) and in the complement factor H deficiency (OMIM #609814), is the most appropriate candidate for genetic inheritance as the encoded protein, i.e. regulatory protein for complement activation of C3 fraction of complement, binds to adrenomedullin, a peptide that belongs to the calcitonin family, eliciting vasodilator effects on the choroid [**12**,**13**]. During pregnancy, CSR patients are more likely to have explicit hereditary history for this disease, either gestational related or spontaneous and is easily detected due to optical coherence tomography (OCT) as an elective diagnostic measure. Most of the examiners would identify a subretinal fluid with yellowish to white fibrinoid exudates in the macular area that might extend to papillomacular areas. In most cases, CSR is reversible in the postpartum period, in weeks to months duration [**12**]. The other proposed mechanisms for CSR association with gestation, include hemodynamic and hormonal alterations, changes in osmotic pressure with a decreased colloid counterpart and hypercoagulability. It is well known that plasma cortisol concentrations are elevated during pregnancy, having their highest levels during the third trimester, when CSR is most likely to occur. The differential diagnosis should include non-arteritic anterior ischemic optic neuropathy, posterior scleritis, and acute retinal necrosis due to the reactivation of Herpes simplex virus and herpes zoster that has been reported during pregnancy [**14**]. 

**Vein and Artery Occlusion in the Retina of Pregnant females**

Pregnancy is a circumstantial factor for the existence of hypercoagulability with visible modifications of platelets, clotting factors along with blood flow hemodynamics. The occlusion of the cilioretinal artery (CRA) is a possible condition encountered in females during gestation, with documented hypercoagulability. The incidence of arterial occlusion first depends on anatomical variations, most of the abnormal arteries arising from the temporal margin of the optic disc, while some of these anomalies may have a nasal aspect origin. Furthermore, the CRA occlusion depends on different quantifiable factors like: coagulation, biochemistry, sedimentation rate, C reactive protein, angiotensin converting enzyme, arterial pressure, homocysteine levels, rheumatoid factor, antinuclear, anticardiolipin, anti-neutrophil cytoplasmic antibodies, together with a positive serology for Rickettsia typhi, Epstein-Barr, varicella virus and cytomegalovirus. The real incidence of CRA obstruction is unknown, probably due to incidence frequency, being most likely during antiphospholipid syndrome when it occurs bilaterally [**15**]. The central retinal vein occlusion (CRV) is less common than CRA occlusion, the condition being mostly associated with PIH, preexisting arteriosclerosis and diabetes. A percent of 51% CRV obstruction female patients were smokers, compared to 40% of those who were diagnosed with CRA occlusion. Furthermore, there is an increase in the cerebrovascular disease and stroke risk, along with a myocardial infarction incidence. It seems that regular exercise and, paradoxically, alcohol intake are a protective factor against CRV occlusion [**16**].

The clinical symptomatology of CRA occlusion may present as a central, homogenous, visual loss that sometimes may occur in the peripheral field, thus, remaining unnoticed, while CRV occlusion may present as a painless monocular vision loss, with a severe prognosis, worse than CRA involvement. Angiography and OCT are essential tools used for the diagnosis in case of retinal vascular obstruction, thus, providing useful data based on measurements of retinal circulation. Indocyanine green angiography is elective in assessing choroidal hemodynamics abnormalities together with hyperviscosity, with a subtle detection of neovascularization that could lead to leakage. The quality of OCT has been improved significantly in the last decade, providing larger resolutions that assure the detection of subretinal fluid as a complication of CRA obstruction. Nevertheless, in the case of a retinal atrophy, OCT helps identify subtle morphological variation in photoreceptors, as a predictive criterion for irreversible visual loss [**17**]. 

**HELLP syndrome**

The HELLP syndrome occurs in 0.1-0.8% of all pregnancies in female patients who suffered from no prior hypertension or proteinuria. The majority of gestations diagnosed with HELLP syndrome developed ascites after cesarean sections with a sudden decrease for platelets count. Furthermore, during post-partum immediate period, preexistent congestive heart failure might aggravate with a six-fold increase for adult respiratory distress syndrome [**18**]. In this context, retinal detachment is an unusual, but detectable phenomenon that occurs in 0.9% of the PIH patients complicated with the HELLP syndrome, being more common in these pathological situations than in preeclampsia and eclampsia alone. Retinal detachment is most likely to be observed during the last trimester of pregnancy, with unilateral serous subretinal fluid accumulation as a consequence of choroidal vascular damage, due to arteriolar vasospasm that would affect the retinal pigment epithelium and subsequent blood retinal barrier impairment, that would latter absorb during the first weeks in the post-partum period. As these situations are considered emergencies, a firm decision of cesarean section with delivery along with a timely ophthalmologic approach would provide the optimal prognosis for any possible visual sequelae [**19**].

**Amniotic fluid embolism**

Although rare, it might become a serious condition with fatal complications during gestation, with a high mortality above 80% accompanied by a noisy clinical tableau that includes shock and convulsions. In this situation, the occipital cortex, visual pathways and optic nerve may become involved with CRA obstruction [**20**].

Thrombotic thrombocytopenic purpura (Moschowitz’s disease or TTP)

Ocular changes are observed in 10% of the rare female patients with TTP, including fundus alterations that include retinal artery narrowing, hemorrhage, serous retinal detachment, and exudates. The most often met visual complaint includes scintillating scotomas, extraocular muscle paresis, and homonymous hemianopia. The ophthalmologist or gynecologist may observe anisocoria and subconjunctival hemorrhage [**21**]. 

**Pituitary apoplexy (Sheehan syndrome)**

Pituitary apoplexy is a pituitary gland enlargement due to the infarction and hemorrhage that occurred in local adenomas that become increasingly frequent in pregnancies, especially as a result of an uncontrolled postpartum hemorrhage. Symptomatology includes sudden headache, complete or as a bitemporal superior quadrant partial vision loss with accompanying ophthalmoplegia, due to cavernous sinus compression and subsequent involvement of the third, fourth and, rarely, the sixth cranial nerves. Therefore, the clinical presentation may evolve to mydriasis anisocoria and diplopia, sometimes, with a full Horner syndrome as a result of sympathetic fibers damage [**22**].

**Disseminated intravascular coagulation**

A frequently observed pathology in a wide range of obstetric complications during pregnancy, occasioned especially by pregnancy toxemia, illegal abortions, or placenta abruption is disseminated intravascular coagulation (DIC). The complication develops as a result of another aggravated, generalized, condition such as sepsis, hemolytic disorder, arterial abnormalities with coaguli formation – hemangioma, prosthetic grafts, autoimmunity – neoplasms, massive trauma. DIC occurs most likely, during gestation, in the context of intrauterine fetal demise, incomplete or septic abortions, PIH or eclampsia [**23**,**24**]. The pathogenesis of DIC relays on tissue factor-mediated initiation of coagulation activation insufficiently balanced by intrinsic physiologic anticoagulant pathways by impaired endogenous fibrinolysis. Furthermore, it seems that microangiopathic hemolytic anemia and vascular endothelial damage via platelet adhesion and activation would facilitate diffuse, generalized, fibrin formation that could facilitate the onset of HELLP syndrome [**25**]. The choroid would be the mostly affected ocular structure in such a situation, increased coagula formation due to continuous platelet consumption and various coagulation factors activation with subsequent fibrinolysis would result in a deranged hemostasis followed by bleeding and widespread ischemia in all organs, thus, resulting in the thrombotic occlusion of CRA and posterior ciliary arteries. Ocular findings would show both eyes with external normal appearance, with delayed filling of the posterior choroid in fluorescein angiography [**26**]. 

## Conclusions

Posterior pole modifications and retinal pathologies that arise during a complicated gestation may pose a serious threat to the mother’s visual acuity along with an impaired quality of life. Furthermore, a vascular caliber reduction in hypertensive disorders, either preexistent or de novo may become a sufficient evidence for maternal-fetal distressed circulation. Ocular symptomatology during pregnancy should not be disconsidered by medical professionals, thus, becoming a clue for an alarming situation that implies both the ophthalmologists and the gynecologists.

**Acknowledgements**

The authors would like to thank the Ophthalmology Department and the Obstetrics and Gynecology Department and their wonderful medical staff both from “Carol Davila” University of Medicine and Pharmacy, Bucharest, Romania and from the University Emergency Hospital, Bucharest, Romania.

**Conflict of interests**

The authors declare no conflict of interest.
